# Aging of Microplastics and Nanoplastics Induced by Advanced Oxidation Processes in Wastewater Treatment and Their Biological Toxicity: A Systematic Review

**DOI:** 10.3390/microorganisms14040812

**Published:** 2026-04-02

**Authors:** Yuxia Li, Lijuan Feng, Shuguang Wang, Chao Song

**Affiliations:** 1 Shandong Key Laboratory of Synergistic Control of Complex Multi-Media Pollution, School of Environmental Science and Engineering, Shandong University, Qingdao 266237, China; 2 College of Geography and Environment, Shandong Normal University, Jinan 250014, China; 3 WeiHai Research Institute of Industrial Technology of Shandong University, Weihai 264209, China; 4 Sino-French Research Institute for Ecology and Environment (ISFREE), School of Environmental Science and Engineering, Shandong University, Qingdao 266237, China

**Keywords:** micro/nanoplastics, UV irradiation, reactive oxygen species, biotoxicity, wastewater treatment, microbial communities

## Abstract

Microplastics and nanoplastics (MNPs), as emerging contaminants, have garnered growing interest for their persistence and biological toxicity. Wastewater treatment plants (WWTPs) are significant convergence points for MNPs, where they undergo complex aging, particularly during advanced oxidation processes (AOPs), leading to different environmental fate and behavior. This study aims to discuss the aging of MNPs in wastewater treatment induced by AOPs and evaluate their biological risks. This review was conducted in accordance with the 2020 PRISMA guidelines. We searched three electronic databases—Scopus, Science Direct, and Web of Science—for relevant articles published between the year 2000 and March 2026. A total of 39 studies met the inclusion criteria and a narrative synthesis was conducted to summarize the findings. Risk of bias assessment was not performed, as this is a narrative systematic review without quantitative synthesis. The review protocol was registered in the OSF (registration DOI: 10.17605/OSF.IO/FTQHN). First, aging pathways and the alterations in the physicochemical properties of MNPs caused by aging are summarized, mainly including changes in surface morphology, crystallinity, and chemical composition, etc. Second, the aging mechanism of MNPs and the factors affecting the aging were discussed. Third, the biotoxicity of aged MNPs on both microorganisms and humans was reviewed, which is mainly due to three sources: plastic particles themselves, released chemicals, and the combination of plastics with coexisting pollutants. Furthermore, this review also criticized the limitations in current studies, the lack of comprehensive evaluation of multiple environmental factors and the identification of specific toxicity; it also provides suggestions for future research. This overview is meaningful for better understanding the environmental fate and risks of MNPs.

## 1. Introduction

Plastic production has been a global industrial activity since the 1940s, with annual consumption increasing significantly in recent decades. Global production reached 400.3 million metric tons in 2023 [1]. Once plastic products lose their intended functionality, they are often discarded, contributing to the growing problem of plastic pollution. Due to their chemical and biological inertness, plastics can persist in the environment for extended periods, which has raised widespread global concern [2]. Wastewater treatment plants (WWTPs) are considered as important sources where MNPs are released into natural waters [3]. In WWTPs, MNPs undergo mechanic wear, chemical oxidation, and biodegradation during various treatments [4]. As a common wastewater treatment process, AOPs utilize highly oxidative substances to decompose organic pollutants. However, this can also partially oxidize MNPs, altering their morphology, mechanical strength, oxygen content, molecular weight, and other properties, thereby influencing their fate and behaviors [5,6]. Therefore, it is critical to understand the changes in physicochemical properties of MNPs in WWTPs and their potential ecological risks.

AOPs, including UV/H_2_O_2_, UV/chlorine, Fenton treatment and persulfate oxidation, are widely applied in wastewater treatment for the removal of recalcitrant organic pollutants [7]. These AOPs generate high concentrations of reactive oxygen species (ROS), such as hydroxyl radicals (•OH), sulfate radicals (SO_4_^•−^), and superoxide anions (O_2_^•−^), which inevitably interact with coexisting MNPs in wastewater, causing the aging of MNPs [8]. During aging, the physicochemical properties of plastics, including color, morphology, size, crystallinity, and hydrophobicity, are altered, leading to the further formation of MNPs and the leaching of plastic additives, both of which pose significant environmental risks [9]. For example, Bárbara Abaroa Pérez et al. found that aged MNPs manifest as surface cracking, yellowing, fragmentation, and structural degradation; these changes subsequently alter their behaviors (e.g., adsorption) [10]. Aged MNPs exhibit changes in specific surface area (SSA) and hydrophobicity, which modify their capacity to adsorb persistent organic pollutants (POPs) and heavy metals [11,12]. Organisms can ingest these contaminant-laden MNPs and trigger adverse physiological responses. Moreover, aged MNPs exhibited biotoxicity on microbial communities during wastewater treatment, such as nitrifying bacteria, denitrifying bacteria, and polyphosphate bacteria, thus disrupting the removal of nitrogen and phosphorus [13]. In addition, the released MNPs in the environment inevitably exposes humans to them, underscoring the urgency of gaining a thorough understanding of their fate in the environment [14,15]. Currently, the understanding of MNPs aging pathways and their negative impacts remain limited. Future efforts should employ multiple analytical techniques to elucidate these processes and conduct in-depth research into how various factors influence aging and the underlying mechanisms. Moreover, the biological consequences of MNPs degradation are still poorly understood.

This review focuses on AOPs-induced MNPs aging and their biological risks to both wastewater treatment technologies and subsequent environmental release. This review aims to: (1) systematically examine the aging of MNPs induced by AOPs, elucidating their mechanisms and governing factors; (2) critically evaluate the biological toxicity of aged MNPs, covering both their intrinsic biological effects and synergistic toxicity with contaminants; (3) identify prevailing knowledge gaps to suggest constructive avenues for future investigations.

## 2. Methods

### 2.1. Literature Identification

This systematic review was conducted following the Preferred Reporting Items for Systematic Reviews and Meta-Analyses (PRISMA) 2020 statement and the “PRISMA_2020_checklist” [16]. The review protocol was retrospectively registered in the Open Science Framework (OSF) to enhance transparency (registration DOI: 10.17605/OSF.IO/FTQHN). This study was not registered in PROSPERO because it does not fall within the scope eligible for registration. There are five steps involved in the process of article retrieval, screening, and analysis: (1) selecting papers through keyword searches; (2) screening titles and abstracts to exclude irrelevant papers based on predefined eligibility and exclusion criteria; (3) full-text screening to summarize the key results of each paper; (4) a detailed analysis focused on the research scale, conceptual framework, indicator composition/classification, and contextual background. We searched papers in three electronic data bases (Scopus, Science Direct, and Web of Science) from 2000 to March 2026 using specific search terms. These databases were chosen to best represent source material in microplastics, nanoplastics, AOPs, aging, and toxicity. To ensure the selected papers are relevant to the aging of MNPs or risk assessment, we grouped the keywords into four categories. The first defines micro/nanoplastics in WWTPs as the study population. The second addresses aging behavior as the targeted. The third highlights the conceptual terms used in assessment studies, and the fourth specifies the research focus and type of literature. The keywords are as follows: (1) microplastics and nanoplastics; (2) aging; (3) wastewater treatment; (4) advanced oxidation processes (AOPs); (5) toxicity. By organizing the keywords into these categories, we ensured that at least one term from each category appeared in the title, abstract, or keywords.

### 2.2. Eligibility and Exclusion Criteria

**Figure 1:**
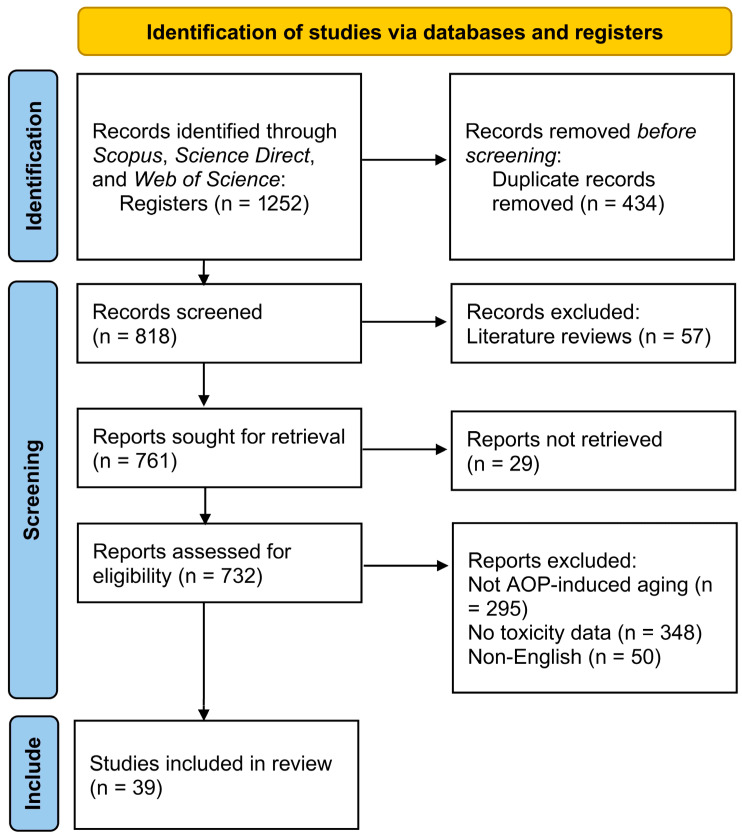
PRISMA 2020 flow diagram for study selection.

We propose the eligibility and exclusion criteria based on three aspects: literature type, research subject, and research content. The literature must consist of empirical studies focused exclusively on the aging of MNPs/risk assessment using multi-source data. The research must examine the impact of MNPs on biological scales. The research content should include clear descriptions of the conceptual framework, data processing methods, and index calculation to ensure rigorous conclusions. The review flow chart is detailed in Figure 1. The initial database search yielded 1252 studies from peer-reviewed journals, including research articles and reviews, from which 434 duplicates were removed. Therefore, we screened the remaining 818 articles and excluded 57 review articles. The remaining 761 articles were screened, and 29 articles for which the original text could not be retrieved were excluded. The titles and abstracts of the remaining 732 studies were independently screened by two reviewers (Y.L. and L.F.) against the eligibility criteria (Table 1). Disagreements were resolved through discussion or by consultation with a third reviewer (C.S.). No automation tools were used in the screening process. A total of 39 articles were selected for full-text review and inclusion. The study selection process is summarized in Figure 1.

**Table 1:** The eligibility and exclusion criteria for choosing articles.

**Literature type**
Eligibility criteria: The article focuses solely on the aging of MNPs/risk assessment studies based on multi-source data. Exclusion criteria: The article is a review, commentary, or conference abstract without original data, or does not focus on the aging process or risk assessment of MNPs.
**Research subject**
Eligibility criteria: The article focuses on the impact of MNPs aging (especially induced by AOPs) on their physicochemical properties, environmental fate, or biological toxicity. Exclusion criteria: The article focuses on the impact of pristine MNPs without considering aging effects.
**Research contents**
Eligibility criteria: (1). The article clearly describes the aging pathways, mechanisms, or factors influencing MNPs under AOPs or environmental conditions. (2). The article investigates the biological toxicity (e.g., to microorganisms, cells, or organisms) of aged MNPs. (3). The article provides detailed data on the characterization (e.g., surface morphology, chemical composition) or toxicological endpoints of aged MNPs.

### 2.3. Challenges in Sampling and Characterization of Nanoplastics (NPs)

While the term MNPs is used throughout this review for brevity, the methods for sampling and characterizing MNPs differ substantially. Microplastics (MPs) in aqueous environments are readily collected using established techniques, such as manta trawls and bongo nets with mesh sizes of 50–500 μm [17]. However, these net-based methods are inherently incapable of retaining NPs due to physical limitations. Sampling NPs requires fundamentally different approaches. Advanced techniques such as cross-flow ultrafiltration (down to 50 nm) [18] and continuous flow centrifugation [19] have been applied to concentrate NPs, though they are technically demanding and not yet standardized. Furthermore, characterization of NPs is equally challenging. Conventional techniques, such as FTIR and Raman spectra, are often insufficient due to diffraction limits and matrix interference [17]. More sophisticated tools, such as pyrolysis–gas chromatography–mass spectrometry, enable polymer identification but provide little information on particle size or number concentration [20]. Emerging techniques, like single-particle ICP-MS and Raman tweezers, offer potential for NP counting and identification, but their application to environmental samples remains in infancy [21]. In summary, the methodological divide between MPs and NPs must be acknowledged. Future studies should explicitly consider these constraints when extrapolating findings from pristine NPs to real-world scenarios.

## 3. Aging Process and Mechanism

### 3.1. Aging Type

In wastewater treatment, MNPs are subjected to aggressive aging induced by AOPs, mainly including UV-based processes (e.g., UV/H_2_O_2_, UV/chlorine, UV/persulfate), Fenton treatment, ozone-based processes (O_3_, O_3_/H_2_O_2_), and persulfate oxidation [7,22,23,24]. AOPs are widely employed to degrade recalcitrant organic pollutants, but they inevitably affect coexisting MNPs, leading to their rapid aging. When polyethylene (PE) (50 mg/L) was exposed to ozone for 8 h, the quality of PE loss was up to 32.56% [25]. Furthermore, Fenton treatment (0.50 mL 20 mM Fe^3+^ and 0.51 mL 30% H_2_O_2_) induced a 60% reduction in the particle size of polystyrene (PS) (from 50 μm to 20 μm) after 108 h, indicating a significant degree of aging [26,27]. Other AOPs, such as O_3_/H_2_O_2_, have also been applied to speed up the aging process of MNPs due to the generation of ROS [28]. Different AOPs exhibit significant variations in their aging efficiencies on MNPs, mainly depending on the oxidants, catalysts, reaction parameters and the physicochemical properties of the MNPs [7].

### 3.2. Physicochemical Property Changes Induced by Aging

Aging induces systematic physicochemical alterations in plastics, including surface morphology (fractures, grooves, cracks, discoloration), hydrophobicity, surface charge, and chemical composition (carboxylation, decomposition) [2]. Due to surface fragmentation and cracks, aging can decrease the particle size and increase the SSA of MNPs. Moreover, there is an obvious negative relationship between particle size and SSA, while small particle sizes due to fragmentation result in high SSA [29]. This phenomenon has been widely observed in different polymer types and aging conditions. For instance, Liu et al. reported that AOPs, such as UV/H_2_O_2_ and UV/Cl_2_ treatment, significantly increased the SSA of PS [30]. Liu et al. [29] reported that aging, induced by Fenton and persulfate treatment for 30 days, led to substantial particle fragmentation in PE, decreasing the average particle size from 45 μm to 9 μm and 17 μm, respectively [29]. Moreover, discoloration is another distinct indicator of MNPs aging. When UV light, oxygen, and other oxidants penetrate the inner layers of MNPs, chromophores are generated or modified, leading to visible discoloration [31]. Furthermore, Fan et al. [32] observed pronounced yellowing in all aged MNPs samples and found that the yellowing intensity was positively correlated with aging time.

Aging may increase the crystallinity of MNPs, largely because of the chain-cracking reactions of amorphous polymers, and thus crystallinity has been selected as a target to evaluate chain cracking in polymers during aging [33]. Furthermore, as the aging time prolongs, the crystallinity of MNPs increases, resulting in more production of fragile MNPs with small particles. For example, the average diameter of polyvinyl chloride (PVC) decreased from 154.11 μm to 119.28 μm after aging. Based on crystallinity calculations, the initial crystallinity of polypropylene (PP) cables was 50.39%, which increased to 67.23% after 16 weeks of aging, reflecting a notable enhancement in crystalline order. In the later stages of aging, a marked increase in the number of smaller MPs was observed [34].

Oxidation, primarily involving carboxylation and the newly formed oxidized products, is an important process in MNPs aging. Therefore, the oxygen-to-carbon (O/C) and carbonyl index (CI) atomic ratios, derived from XPS and FTIR data respectively, also serve as the indicators of MNPs aging, resulting from the positive correlation between them and aging time [29,35,36]. Furthermore, oxidation can alter the surface charge and hydrophilicity of MNPs due to the generation of oxygen-containing functional groups. In general, aged MNPs exhibit stronger electronegativity and more hydrophilicity [32,37,38].

### 3.3. Aging Mechanism

**Figure 2:**
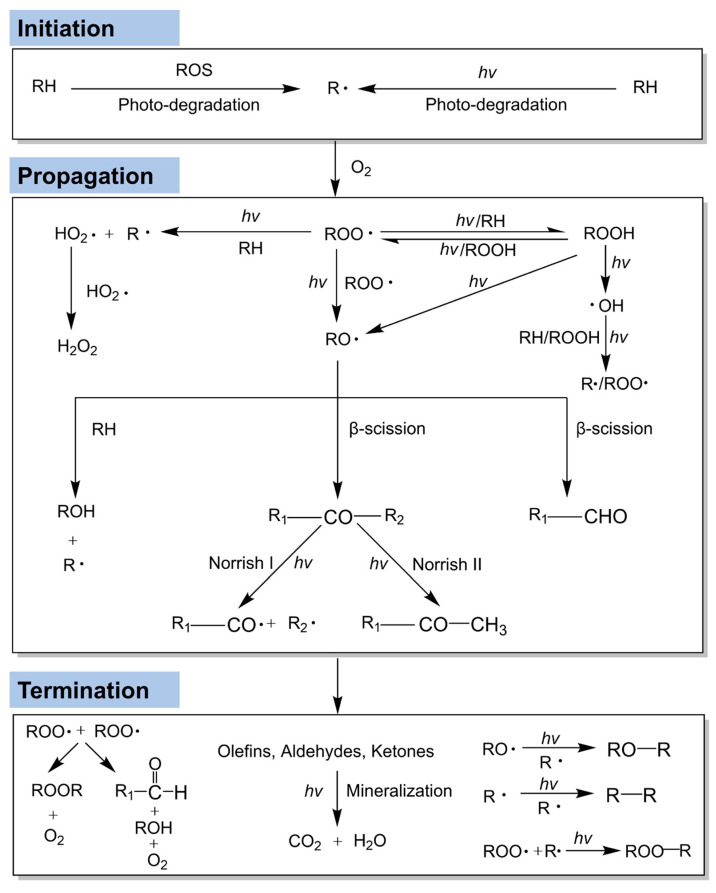
The aging mechanism of MNPs induced by UV irradiation. RH stands for MNPs, and R_1_ and R_2_ indicate different polymer chains of variable lengths. This figure was adapted with permission from reference [40].

AOPs-mediated aging of MNPs is primarily induced by UV radiation and ROS [7,23]. UV exposure can induce embrittlement and surface cracking in MNPs and accelerate the generation of free radicals that further promote their aging process (Figure 2) [39]. The aging of MNPs typically proceeds through initiation, propagation, and termination. UV irradiation initiates the process by exciting chromophores (e.g., phenyl rings in PS or impurities in PE), which absorb photons to generate excited singlet states. These states undergo intersystem crossing to triplet states, enabling energy transfer to adjacent C–H or C–C bonds and subsequent formation of alkyl radicals (R•). The rapid reaction of R• with atmospheric oxygen yields peroxy radicals (ROO•), marking the onset of oxidation. The propagation phase amplifies oxidative damage through autoxidation. ROO• abstracts hydrogen atoms from the polymer backbones, forming hydroperoxides, which then decompose under UV irradiation to yield alkoxy radicals (RO•) and •OH. The RO• radical, a highly reactive and light-sensitive intermediate, may abstract hydrogen from polymer chains to generate alcohols or undergo β-scission to form ketones and aldehydes. These secondary carbonyl products are subsequently oxidized to carboxylic acids and esters. In the termination phase, reactive radicals react with each other or with stabilizing agents present in the polymer matrix, forming stable, nonradical products and thereby completing the aging process [40].

**Figure 3:**
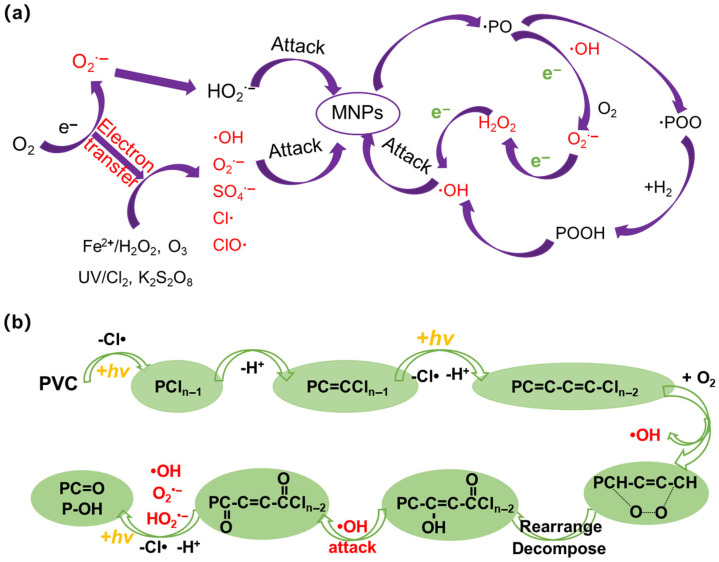
(**a**) The aging mechanism of polyolefin MNPs induced by AOPs (excluding UV radiation) in wastewater treatment. (**b**) The aging process of PVC MNPs in wastewater treatment.

UV light serves as an activator for oxidants such as H_2_O_2_, chlorine, or persulfate, generating high fluxes of ROS that attack the polymer chains of MNPs [7,41]. ROS include •OH, O_2_^•−^, H_2_O_2_, and other free radicals, such as SO_4_^•−^, Cl• and ClO• (reactive chlorine species, RCS) [42,43]. Since •OH is non-selective with a high redox potential (2.8 V) [44], and leads to the formation of other ROS [43], to some extent it is considered the main factor for AOPs-induced aging of MNPs. AOPs rely on externally added oxidants/catalysts to generate high-flux, dominant •OH radicals under energy input, aiming to achieve rapid mineralization of pollutants (Figure 3a). Due to the high-concentration of ROS in AOPs, MNPs undergo rapid aging and even degradation via polymer chain scission and oxidative fragmentation into low-molecular-weight products [37]. For example, the dechlorination of PVC was up to 75% within 6 h in the Fenton system [45].

Compared to polyolefin MNPs, the aging of halogen-containing plastics involves dehalogenation [45]. Taking PVC as an example, the polymer undergoes cleavage of its C–Cl bond upon absorbing energy from AOPs (Figure 3b), forming a polyene structure (represented as PCl_n−1_). This structure can subsequently undergo further reactions [46]. The processes of C–Cl bond cleavage and H-atom abstraction proceed continuously in a chain reaction. During dechlorination, the resulting intermediates react with O_2_ to form •OH radicals and peroxyl radicals (P–OO•). The subsequent decomposition and rearrangement of the polymer backbone then lead to the formation of -OH [47]. The generated •OH radicals can subsequently attack the polymer backbone, abstracting a hydrogen atom to form C=O on the PVC chain [48]. During the aging process, the above reactions, including hydrogen abstraction, C-C and C-Cl bond cleavage, and •OH attack, proceed continuously. In addition, some metal oxides, such as InVO_4_/BiOBr and TiO_2_, are commonly added to plastics as additives, and they can work as photocatalysts to promote the aging of MNPs [49,50].

### 3.4. Factors Affecting the Aging Process

**Figure 4:**
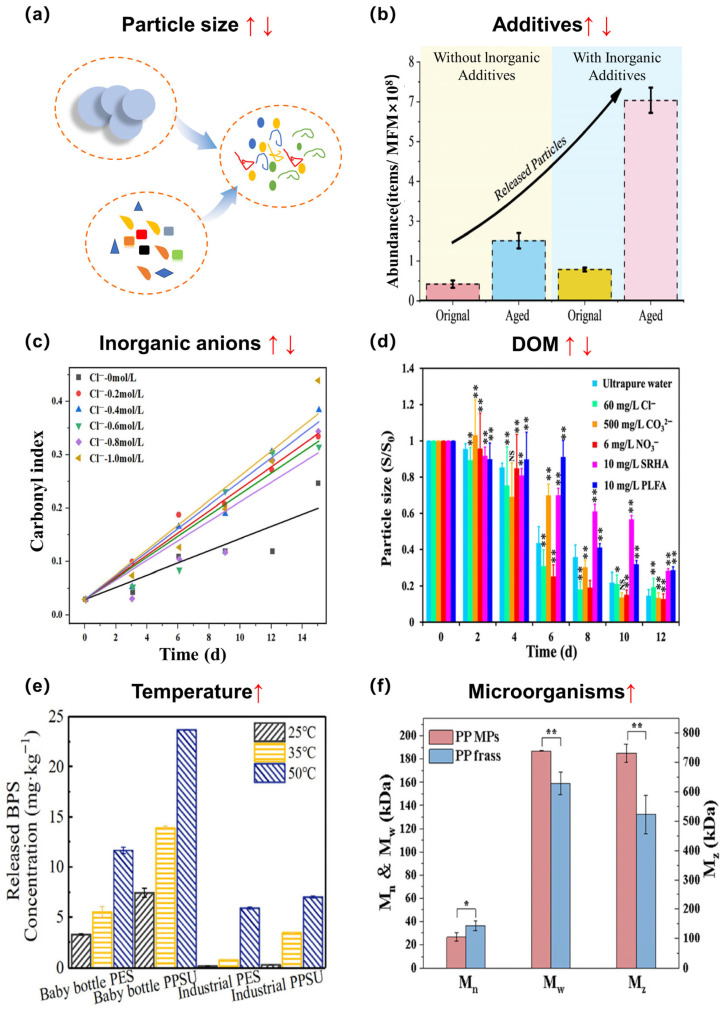
Factors influencing the aging of MNPs, including material properties and environmental conditions that drive physicochemical and biological transformations. An upward arrow indicates that the factor promotes plastic aging and a downward arrow indicates that the factor inhibits plastic aging, while the simultaneous presence of both arrows signifies that the factor has multiple effects on plastic aging. Statistical analysis was performed using one-way ANOVA. * *p* < 0.05 and ** *p* < 0.01 were considered as statistically significant differences compared to the control group. This figure was adapted with permission from references [60,65,66,67,68,69].

The aging of MNPs in wastewater treatment is commonly driven by various factors, such as particle size, additives, inorganic ions, dissolved organic matter (DOM), temperature, and microorganisms (Figure 4). The particle size of plastic debris plays complex roles in the aging of MNPs. Pre-existing smaller particles, caused by aging-induced fragmentation or initially smaller particle size, generally exhibit higher reactivity due to their increased SSA, which creates more reactive sites, and drives radical-mediated oxidation processes [8,51]. PP with sizes of 0–150 μm and 300–500 μm showed faster oxidation than those spanning 1–2 mm and 5–7 mm, mainly due to their higher SSA, enabling greater UV energy capture and subsequent •OH production in wastewater [51]. For PVC, the aging process also follows this size-reactivity correlation, with smaller PVC particles degrading more rapidly under UV exposure in the presence of organic acids, driven by increased free radical activity [47]. Meanwhile, aging also causes plastic particles to shrink in size, affecting related physicochemical properties and thereby accelerating subsequent aging processes [7]. Therefore, the influence of particle size on plastic aging is closely related to multiple physicochemical properties of plastics and should be assessed comprehensively, rather than as a single factor.

Inorganic ions, especially anions, mainly affect the aging of MNPs by participating in the generation of ROS [37]. Halide ions (e.g., Cl^−^, Br^−^) significantly accelerate or inhibit the aging of MNPs, resulting from the interaction with •OH to generate reactive halogen species (Cl•, Br•, Cl_2_^•−^, Br_2_^•−^, and ClBr^•−^, etc.) [52,53]. These secondary radicals also exhibit strong oxidative potential, thereby leading to polymer chain scission and surface oxidation of MNPs during aging. Wu et al. [37] found that Cl^−^ was converted into Cl_2_^•−^ and prevented the generation of O_2_^•−^, resulting in the slower aging rate of PP in seawater than that in ultrapure water. Nitrate can be excited by UV irradiation to generate highly reactive species such as •OH, which significantly accelerates the MNPs’ aging [54]. In contrast, carbonate and bicarbonate can quench •OH to form a carbonate radical (CO_3_^•−^), which exhibits high steady-state concentration and long lifetime [55,56]. However, CO_3_^•−^ primarily oxidizes electron-rich compounds (e.g., nitrogen- or sulfur-containing moieties) due to its relatively low oxidation potential (E = 1.59 V vs. NHE) [57], and thus carbonate and bicarbonate generally inhibit the aging of most MNPs. However, Zhu et al. [54] found the negligible effect of HCO_3_^−^ on PS aging, due to the oxidation of electron-rich benzene rings in PS by CO_3_^•−^. These contrasting results indicate that the types of MNPs also play a crucial role in the aging process.

Given the ubiquitous presence of DOM in the wastewater, it is commonly employed as a key factor to influence the MNPs’ aging [58]. In general, DOM play dual roles in the aging of MNPs as both an inhibitor and an accelerator. Specifically, both humic acid (HA) and fulvic acid (FA) can effectively scavenge ROS, thus suppressing their availability and weakening their interaction with MNPs [59,60]. The abundant functional groups of DOM can attach to MNPs’ surfaces via hydrophobic or electrostatic forces, subsequently influencing aging efficiency by either occupying reactive sites or shielding against photon exposure [60,61,62]. Moreover, DOM could accelerate the aging of MNPs, mainly depending on their molecular weight, structure and redox property. Compared to DOM at higher molecular weight (e.g., >5–30 kDa), DOM at low molecular weight (<1 kDa), with abundant fluorescent moieties, would facilitate ROS generation under UV radiation and exhibit superior electron-donating capacity, thereby enhancing the aging of MNPs [63]. Also, the hydrophilic fraction of DOM, rich in tannin-like constituents and carboxylic acids, exhibits optimal electron-accepting capacity and oxidation properties, which could enhance ROS generation, particularly •OH, thereby promoting the aging of MNPs [64].

Temperature influences both the abiotic and biotic aging processes of MNPs. Briefly, temperature changes the bond energy of polymer chains in MNPs, accelerating physical degradation (e.g., chain scission) and chemical reactions (e.g., oxidation) [70]. High temperatures promote thermal decomposition of aging-induced peroxides to generate free radicals, drive polymer chain scission, and compromise surface mechanical properties, thereby enhancing molecular mobility and facilitating the leaching of oligomers and additives [71,72]. Moreover, temperature modulates the biotic aging process by altering microbial community and regulating metabolic activity (e.g., enzyme production).

Additives released during plastic aging can influence the overall aging process of MNPs [73]. Wang et al. found that inorganic additives can substantially accelerate the aging of plastic through multiple mechanisms, leading to increased generation of small-sized MNPs [65]. In addition, additives played important roles in the complex and dynamic “feedback loop” between plastic aging and biofilms. The initial colonization by microorganisms and subsequent biofilm formation accelerates plastic aging through biochemical and physical processes [74,75]. This aging, in turn, alters the plastic’s surface properties, such as increasing roughness and hydrophobicity, thus facilitating further microbial adhesion [7]. A critical, self-reinforcing component of this loop is the aging-induced release of chemical additives and the generation of dissolved organic matters [76]. These released substances can serve as a nutrient source, promoting microbial growth and metabolic activity, which then intensifies the degradation process [7]. Moreover, it is crucial to recognize the dual role of additives. Some additives, such as plasticizers or antimicrobial agents, may exhibit toxicity that can inhibit microbial activity or reduce biofilm adhesion, thereby introducing a potential negative feedback mechanism into the system [77]. Therefore, additives can also affect plastic aging via regulating biofilms. In this regulation, microorganisms also play dual roles: they accelerate plastic aging through biofilm formation, and their community structure and function are in turn disrupted by aged MNPs (Details were discussed in Section 4.1).

In wastewater treatment, the aging of plastics arises from the combined effects of physical, chemical, biological processes, involving multiple factors. However, most current studies on MNPs aging have been conducted under simulation conditions in laboratories, which differ from actual environmental conditions [1,78]. Therefore, most plastic aging simulations in laboratories lack environmental relevance, making it difficult to accurately assess the fate of plastics in real-world environments. Future studies should prioritize the use of UV intensities and oxidant concentrations consistent with real wastewater treatment, while comprehensively considering the synergistic effects of multiple factors to more accurately assess the fate of MNPs during wastewater treatment. Furthermore, these physicochemical alterations induced by AOPs—particularly the reduction in particle size, increase in specific surface area, generation of oxygen-containing functional groups, and accelerated release of additives—have profound implications for the environmental fate and biological effects of MNPs. These changes enhance the bioavailability of MNPs, facilitate their transport across biological barriers, and increase their toxicity to organisms across different biological levels, from microbial communities to human health. Therefore, it is essential to understand these aging-induced transformations for assessing the potential health risks associated with MNPs released from WWTPs.

## 4. Biological Risks of Aged MNPs

### 4.1. Effects of Aged MNPs on Microorganisms in WWTPs

Microorganisms are the first biological level affected by MNPs in WWTPs. Activated sludge, which harbors diverse microbial communities responsible for pollutant removal, retains approximately 90% of MNPs from wastewater streams [79]. The accumulated MNPs can adversely affect nitrogen and phosphorus removal by disrupting key microflora. For instance, He et al. [13] reported that polystyrene MNPs at 0.1 g/L inhibited ammonia oxidation by 71%, via affecting both ammonia-oxidizing and nitrite-oxidizing bacteria. Similarly, aged polyvinyl chloride MNPs have been shown to severely inhibit the removal of NH_4_^+^-N, accompanied by reduced sludge concentrations [80]. Similar to pristine MNPs, aged MNPs can also affect the physiological processes of microorganisms and exhibit stronger biotoxicity due to their increased specific surface area, rougher surface morphology, and enhanced hydrophilicity, and they also exhibit stronger interactions with sludge flocs, therefore promoting their accumulation in the sludge [6,13,81]. Once released from WWTPs, these plastic particles can also exhibit biotoxicity on microorganisms in nature. For example, Lang et al. found that PS MNPs altered eukaryotic and heterotrophic bacterial communities [82]. In addition, the intracellular content of microalgae (i.e., protein and carbohydrate levels) continued to decline after exposure to MNPs, and this inhibition was even more pronounced when aged PVC was used [1].

### 4.2. Cellular Toxicity

**Figure 5:**
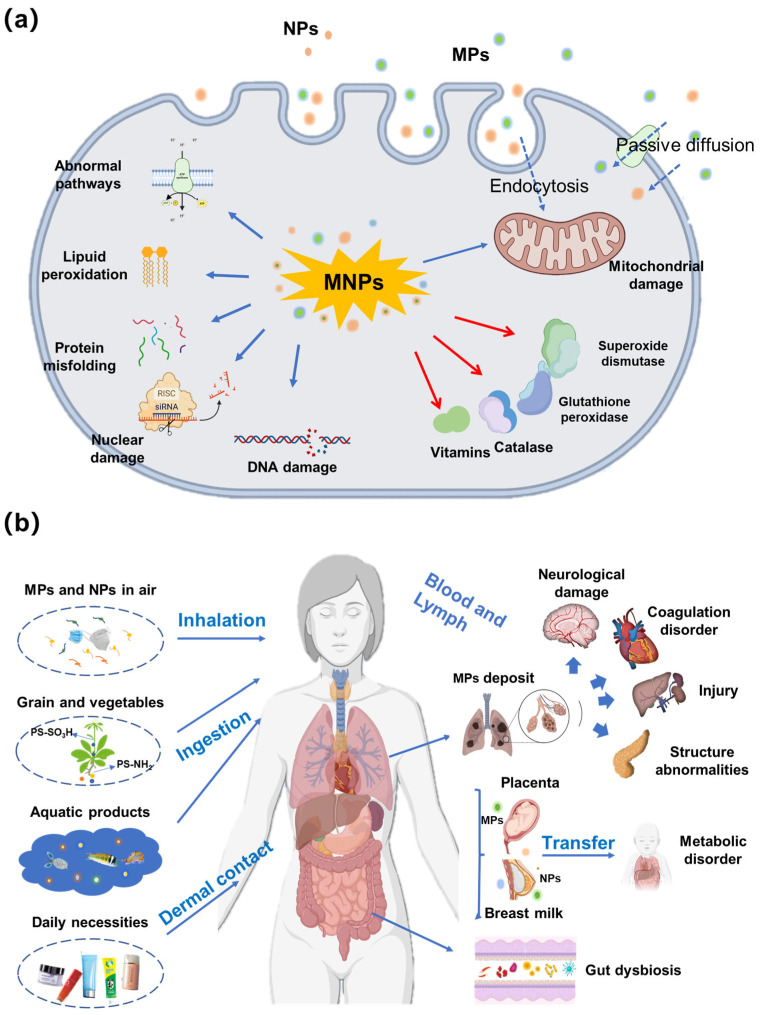
The inherent toxicity of MNPs at the cellular (**a**) and organismal (**b**) levels. MNPs cause channel dysfunction, lipid peroxidation, protein misfolding, and nuclear damage. MNPs enter the human body through inhalation, ingestion, and dermal contact, causing neurological damage, coagulation disorders, and other damages.

At the cellular level (Figure 5a), MNPs can be internalized through endocytosis or passive transport, and their subcellular localization is size-dependent. While larger particles tend to accumulate in the cytoplasm, smaller MNPs (~30 nm) may even translocate into the nucleus and interfere with chromatin structure and function [83,84]. Internalization of MNPs triggers intracellular ROS generation, leading to oxidative stress [85]. Notably, this oxidative response is more pronounced with aged MNPs. Pannetier et al. [86] observed higher ROS production in fish exposed to weathered MPs, compared to virgin MPs, attributing to the additional contaminants adsorbed onto aged particles. Prolonged oxidative stress can overwhelm cellular antioxidant defenses, as evidenced by decreased expression of superoxide dismutase and catalase following MNPs exposure [87]. This oxidative environment inflicts damage upon multiple cellular components, including lipid peroxidation, protein misfolding, mitochondrial dysfunction, and DNA damage [23]. Exposure to MNPs consistently results in reduced cell viability and increased cytotoxicity across various cell types. For example, a corresponding decrease in BCL-2 expression in HK-2 human kidney cells was observed, accounting for reduced cell viability [83].

### 4.3. Tissues and Organs Toxicity

MNPs have been detected in various organs of aquatic organisms and mammals (Figure 5b), where they induce structural and functional damage. In fish, accumulation of MNPs has been observed in the gills, liver, and intestines, leading to tissue inflammation, histological alterations, and impaired organ function [88,89]. For instance, co-exposure to MNPs and polybrominated diphenyl ethers exacerbated hepatotoxicity in zebrafish, manifesting as hepatic discoloration, liver atrophy, and altered embryonic development [89]. In mammalian models, MNPs have been shown to accumulate in the liver, kidneys, and lungs, where they trigger inflammatory responses and tissue damage [90]. For example, microplastics were detected in all 17 arterial samples, with an average concentration of 118.66 ± 53.87 μg/g tissue. The concentrations of microplastics in arteries containing atherosclerotic plaques, both coronary arteries (156.50 ± 42.14 μg/g tissue), and carotid arteries (133.37 ± 60.52 μg/g tissue), were significantly higher than that in aortas without atherosclerotic plaques (76.26 ± 14.86 and 76.26 ± 14.86 μg/g tissue), suggesting that microplastics might be associated with atherosclerosis in humans [91]. The small intestine is particularly vulnerable, as MNPs can be taken up by intestinal epithelial cells, potentially disrupting nutrient absorption and gut barrier function [92]. PS-MPs can also induce inflammatory injuries in multiple murine organs via the TLRs/MyD88/NF-κB pathway [93].

### 4.4. Human Health Risk

A significant proportion of MNPs released into the environment originate from WWTPs, where they undergo accelerated aging through AOPs [3]. Aged MNPs, with their altered physicochemical properties, such as smaller particle size, rougher surface, and higher surface reactivity, enter the environment through effluent discharge or sludge application [7]. The widespread presence of MNPs inevitably leads to human exposure through multiple pathways, including the ingestion of contaminated food and water, inhalation of airborne particles, and dermal contact [94,95,96]. Compared to pristine MNPs, aged particles may pose greater health risks due to their enhanced ability to penetrate biological barriers, carry co-existing pollutants, and induce oxidative stress and inflammation [83]. Specifically, MNPs have been detected in various human samples (Figure 5b), including saliva, breast milk, lungs, liver [88], kidneys [92], colon and blood [97]. Inhaled MNPs can penetrate deep into lung tissues, potentially causing cytotoxicity and inflammatory responses in pulmonary epithelial cells [98]. In the gastrointestinal tract, ingestion of MNPs through contaminated food and water may lead to accumulation and adverse effects on digestive health [87]. Emerging evidence suggests that MNPs may cross the blood–brain barrier and induce neuroinflammation, potentially contributing to neurodegenerative diseases [99]. Furthermore, MNPs have also been detected in the human cardiovascular system, raising concerns about their potential role in cardiovascular diseases [100]. Transgenerational impact of MNPs should be of particular concern, as plastic particles can penetrate the placental barrier and reach the fetus [92]. Long-term exposure to aged MNPs may cause chronic oxidative stress and inflammation, which are associated with various human diseases, including pulmonary disorders, cardiovascular diseases, and cancer [87].

### 4.5. Toxicity Mechanism of Aged MNPs

The biological toxicity of aged MNPs is mainly attributed three sources: the toxicity of the particles themselves, the toxicity of chemicals released from MNPs—such as additives—and the combined toxicity of plastics with coexisting pollutants.

#### 4.5.1. The Inherent Toxicity of MNPs

**Figure 6:**
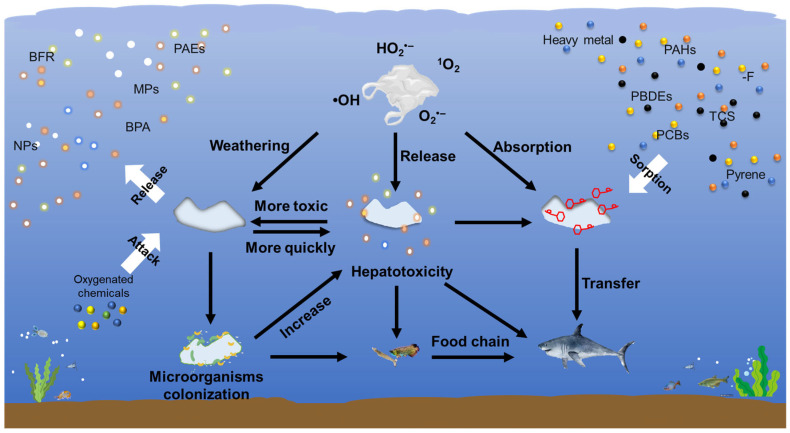
Biological risks of MNPs. Aged plastics release additives such as BPRs, PAEs, and BPA. MNPs mainly act as carriers for various hazardous substances, triggered multifaceted toxicity.

Microplastic ingestion, or interaction in multiple ways, has been reported in a variety of aquatic organisms such as planktons, aquatic plants, invertebrates, fish, waterbirds and other top predators (Figure 6). The degree of microplastic degradation affects the uptake of MNPs by different aquatic organisms. Trophic transfer can serve as an indirect approach of microplastic uptake by different trophic level predators [101]. Additionally, biofilms on plastic surfaces influence microplastic ingestion [102].

The toxicities of MNPs have been reported on bacteria, fungi, archaea, phytoplankton, animals, and even humans, and the details are listed in Table 2. The biological toxicity of MNPs is predominantly determined by their physicochemical characteristics, including particle size, dosage, exposure duration, polymer composition, and surface hydrophobicity, most of which are dynamically altered during aging processes [1,5,103,104]. Notably, aged MNPs exhibit enhanced bioavailability to organisms and demonstrate distinct toxicological profiles compared to their pristine counterparts. For example, aging treatment increased the cytotoxicity of phenol-formaldehyde resin MNPs in A549 human lung adenocarcinoma cells, which was attributed to the elevation of their oxidation potential [105]. In addition, aged MNPs significantly amplify oxidative stress and metabolic disruption. Wang et al. [106] reported that both pristine and aged PS triggered substantial ROS generation in *Drosophila salina*, leading to metabolic reprogramming in algal cells (Table 2). Similarly, Liu et al. [27] observed that aged tire wear particles exhibited enhanced toxicity and caused macrophage damage, primarily attributed to ROS generation induced by environmentally persistent free radicals formed during aging.

**Table 2:** The reported biological toxicity of MNPs in published papers.

Polymer Type	Exposure Dose	Exposure Time	Toxicity	Reference
PS-MPs (5 μm), Imidacloprid	PS-MPs: 20 μg/L Imidacloprid: 100 μg/L	21 days	Zebrafish: inhibited growth, greater changes in expression of glycolipid metabolism and inflammation-related genes	[107]
PS MPs (2.6 μm)	1 × 10^5^ μg/L	5 days	*Dunaliella salina*: inhibited growth, oxidative damage, upregulated amino acid-related metabolic pathways, inhibited cell division	[106]
PS (1.7 μm)	10 μg/cm^2^	2 days	human lung epithelial (BEAS-2B): cytotoxicity, round shape, shrink	[108]
PS-NPs (50 nm), Bisphenol A (BPA)	PS-NPs: 1000 μg/L BPA: 1 μg/L	1 day	Zebrafish: BPA in the viscera (85 μg/g *ww*), gill (43 μg/g *ww*), head (20 μg/g *ww*), and muscle (20 μg/g *ww*)	[109]
PS-NPs (80 nm), Brominated Diphenyl Ether-47 (BDE-47)	PS-NPs: 1 × 10^4^ μg/L BDE-47:10 μg/L	1 day	Zebrafish juveniles: oxidative stress, hepatotoxicity, and neurodevelopmental toxicity	[89]
PP-MPs (<150 μm), Cd	PP-MPs: 9000 mg/kg Cd: 8.1 mg/kg	42 days	*Eisenia foetida*: values of 4.3–67.2 particles/g of MPs in earthworm, the accumulation of Cd in earthworm (161.3%)	[110]
PE-MPs (250–300 μm)	4 × 10^5^ μg/L	3 days	*Vibrio fischeri*: growth inhibition of 27%	[111]
Tire wear particles MPs (150 μm)	1 × 10^6^ μg/L	1 day	Macrophages: decrease in cell viability (27–45%), increase in oxidative stress response (46–93%), and inflammatory factor secretion	[27]

For most living species, MNPs can enter the cells through passive transport or endocytosis. They can accumulate within the mitochondria, further damaging the electron transport chain, causing membrane damage, disrupting membrane potential, or leading to mitochondrial depolarization, ultimately resulting in the production of ROS [88]. These free radicals can induce lipid peroxidation, protein oxidation, DNA damage, and disruption of the antioxidant defense system [112]. The interaction between immune cells and plastic particles, or the cell damage mediated by oxidative stress, may result in the promotion of pro-inflammatory cytokines. Such inflammation is a hallmark of cardiovascular, neurodegenerative, and other age-related disorders [83].

Few studies concluded the opposite results that oxidation could alleviate the toxicity of MNPs to organisms [113,114]. Zou et al. indicated that pristine small-sized PA MPs (8.13 μm) with a high aggregation potential tended to accumulate in organisms, while aged ones with higher stability were easier for zebrafish larvae to excrete [113]. Such a distinct result may also be related to the larger particle size of pristine MNPs, which were easily intercepted by the intestinal villi and difficult to be excreted directly by the larvae [115].

#### 4.5.2. Toxicity of Released Chemicals

A significant proportion of MNPs persist in aquatic and terrestrial environments, where they undergo aging via physical, chemical, and biological processes. These transformations lead to the leaching of short-chain organic compounds, including volatile organic compounds (VOCs) and DOM. A diverse range of degradation products (alcohols, ketones, aldehydes, etc.), carboxylic acids, and fruit acids have been identified in the soluble fractions of aged MNPs [116]. Zhou et al. [117] explored the leaching behavior of organic compounds from MNPs subjected to 120 min of plasma treatment. They found that the dissolved organic carbon released from PE, PS, PVC, and PP was 14.0, 5.09, 4.33, and 4.35 mg/L, respectively. Furthermore, the release of DOM and VOCs enhanced the toxicity of aged MNPs. Xu et al. [74] reported that the concentration of polyurethane-derived DOM rose with prolonged aging, correlating with enhanced cytotoxicity in human colon adenocarcinoma cells. Wu et al. found that dibutyl phthalate and 4-acetylbenzoate, VOCs with high potential toxicity, were released from PE and PET during UV irradiation, respectively [104].

Furthermore, aging promotes the release of additives and leachate from MNPs, enhancing the toxicity and risk of MNPs in nature. Commercial plastics typically contain diverse additives, such as phthalates (PAEs) [77,118], brominated flame retardants (BFRs) [119], BPA [120], heavy metals [121], and other organic compounds, which are used to enhance plastic properties and durability. However, owing to their physical blending rather than chemical bonding within the polymer matrix, these additives are highly mobile and can leach out from MNPs into the environment during the aging process (Figure 6). Generally, aging accelerates additive release through two primary mechanisms: First, the formation of surface cracks and fragmentation in aged MNPs significantly increases their contact area with oxygen-rich compounds or aqueous solutions, facilitating additive diffusion [76]. Second, polymer chain scission induced by aging disrupts the structural integrity of plastics, thereby liberating entrapped additives [33,76]. These synergistic effects collectively amplify the environmental hazards posed by aged MNPs compared to their pristine counterparts. Several studies have quantified the leaching behavior of plastic additives under various aging conditions. For example, PAEs were considered as the predominant additives released from two common plastic materials, cable insulation and plastic garbage bags, during aging [77]. After exposure to UV irradiation for 90 days, diethyl phthalate and dimethyl phthalate were the dominant released PAEs from cable insulation, reaching maximum concentrations of 68.9 ± 10.3 ng/g and 9.5 ± 1.4 ng/g, respectively [77]. Similarly, plastic garbage bags primarily leached DnBP and diisobutyl phthalate, with peak concentrations of 120.1 ± 18.0 ng/g and 83.4 ± 12.5 ng/g, respectively [77]. Besides PAEs, other additives also exhibit accelerated leaching from aged MNPs. Luo et al. [76] found that aged MNPs released TiO_2_ nanoparticles more rapidly and in greater quantities than pristine MNPs. Acidic conditions also exacerbate lead chromate leaching from MNPs, with prolonged aging intensifying its toxicity [122]. Furthermore, UV radiation is a key driver of additive release. For example, 24 h UV exposure significantly increased the leaching of organotin stabilizers, including dimethyltin, monomethyltin, dibutyltin, and monobutyltin, from PVC into aqueous environments [123]. Similarly, the aging of PS MNPs facilitated the release of brominated flame retardants, such as tetrabromobisphenol A bis(2,3-dibromopropyl ether), tetrabromobisphenol A (TBBPA), and decabromodiphenyl ether, into both water and air [124]. The leaching of additives and plastic-derived DOM can directly affect planktonic microbial growth and alter microbial community composition in receiving waters [74].

#### 4.5.3. Combined Toxicity of MNPs with Co-Existing Contaminants

The biological risks posed by MNPs extend beyond their intrinsic toxicity, as they frequently interact with other environmental contaminants to induce synergistic or additive toxic effects (Figure 6). During these interactions, MNPs mainly act as carriers for various hazardous substances, thus promoting the adsorption and ingestion of co-existing contaminants by organisms [84]. Due to the high toxicity of heavy metals and organic pollutants associated with MNPs, it is crucial to investigate their interaction mechanisms with co-existing contaminants to better assess their biological risks.

Aging enhances the interactions between MNPs and contaminants by increasing their SSA, imparting a more hydrophilic surface and generating more active sites (e.g., oxygen-containing groups). Specifically, more oxygen-containing functional groups are generated on MNPs surface during aging, resulting in the surface being more negatively charged. The surface charge of MNPs, significantly altered by aging, determines the extent of electrostatic attraction or repulsion to other contaminants. Electrostatic interactions (π-π interactions and hydrogen bonding) play a critical role in the adsorption of charged pollutants, including heavy metals (e.g., Pb^2+^, Cu^2+^) and ionizable organic contaminants [125]. Moreover, biofilm has also been considered an important factor affecting the adsorption of heavy metals onto aged MNPs [25]. Biofilm could accelerate the aging of MNPs by modifying their surface properties, such as hydrophobicity and surface charge, which in turn alters their heavy metal adsorption capacity [126].

MNPs exhibit a strong affinity for hydrophobic organic pollutants, such as polychlorinated biphenyls [109,127], endocrine-disrupting chemicals [128,129], and polycyclic aromatic hydrocarbons (PAHs) [130,131,132]. Avio et al. [130,133] reported that PE and PS MNPs can adsorb pyrene, enhance its bioavailability, and thus lead to bioaccumulation in digestive tissues, hemolymph, and gills of mussels, which triggers multifaceted toxicity, including immunosuppression, lysosomal membrane destabilization, peroxisome dysfunction, and neurotoxicity. In addition, Wang et al. [89] reported that MNPs facilitated polybrominated diphenyl ethers uptake in zebrafish, amplifying neurodevelopmental toxicity and hepatotoxicity, such as hepatic discoloration, liver atrophy, and altered embryonic development. Likewise, co-exposure to MNPs and the neonicotinoid insecticide imidacloprid exacerbated hepatic inflammation in zebrafish, underscoring the role of MNPs in potentiating chemical toxicity [107]. In addition, MNPs can also enter the food chain and act as carriers of pollutants, posing a threat to wild animals [134,135,136].

In terrestrial ecosystems, MNPs interact with agrochemicals and pharmaceuticals, exacerbating their biological impacts. For example, PE MNPs combined with herbicides (e.g., simazine) or pharmaceuticals (e.g., ibuprofen) induced greater toxicity in *Caenorhabditis elegans* than MNPs alone [111]. Moreover, MNPs also exhibit high adsorption capacity for heavy metals (e.g., Pb, Cd, Cu), which can be rapidly acquired from environmental sources, such as antifouling paints [137]. Due to their long-term retention and high bioavailability, metal-laden MNPs induce chronic toxicity in aquatic organisms, including reproductive interference, intestinal dysbiosis, and cytotoxic damage [137,138,139,140]. For example, it has been reported that the association between toxic heavy metal ions and MNPs can exacerbate autophagy, oxidative stress, fibrosis, and cell apoptosis in marine organisms [141,142]. Furthermore, MNPs can also significantly enhance the bioaccumulation of some organic pollutants and heavy metals. For instance, PS MNPs increased BPA accumulation in the viscera and head of zebrafish by 2.2 and 2.6 times after 3-day exposure, respectively [109]. Similarly, the accumulation of cadmium in *Eisenia fetida* increased by 161.3% in the presence of PP after 42-day combined exposure [110].

Overall, the toxicity of aged MNPs mainly results from these sources: plastic particles themselves, released chemical from MNPs, and the combination with other coexisting pollutants. Therefore, it is essential to identify the specific reasons to fully explore the toxicity of MNPs. This is also another limitation of some current studies on the toxicity of aged plastics. Isolating the aged particles from leachate before being introduced to the organism is suggested to evaluate the inherent toxicity of aged MNPs, and the combined toxicity of MNPs with co-existing contaminants should also be fully evaluated in future studies. Notably, it is important to recognize that the three toxicity mechanisms are not independent during the aging process. Aging, particularly induced by AOPs, exacerbates each of these pathways: (i) it increases the inherent toxicity of MNPs by reducing particle size and enhancing surface reactivity, facilitating cellular uptake and oxidative stress; (ii) it accelerates the release of toxic additives such as phthalates, BPA, and flame retardants; and (iii) it strengthens the adsorption capacity for co-existing pollutants, enhancing their bioavailability and combined toxicity. Therefore, the health risks associated with MNPs should be evaluated in the context of their environmental aging history, especially in WWTPs where AOPs are applied.

### 4.6. Modeling of MNPs Effects on Living Organisms

Modeling approaches have emerged as powerful tools to predict and understand the toxicity of MNPs across different biological scales, complementing experimental studies and reducing the need for animal testing [143]. Molecular dynamics simulations investigate MNPs interactions with cellular membranes at the atomic level. These simulations reveal that MNPs can penetrate lipid bilayers, induce membrane disruption and pore formation, with smaller and positively charged MNPs exhibiting greater membrane damage [144]. Toxicokinetic-toxicodynamic models predict MNPs absorption, distribution, metabolism, and excretion and link internal concentrations to adverse effects over time [145]. Such models have been developed for organisms like *Daphnia magna* and zebrafish to estimate key parameters, such as LC_50_ and no-effect concentrations, enabling extrapolation from acute laboratory exposures to chronic environmental scenarios. Protein corona modeling addresses how plastic particles acquire a biological identity upon entering physiological fluids [146]. Computational approaches predict corona composition and its effects on cellular uptake and toxicity, revealing that corona formation can either enhance or mitigate the toxicity of MNPs depending on the adsorbed proteins. Environmental fate models [147] (e.g., FRAGMENT-MNPs, FLEXPART) simulate the fragmentation, transport, and distribution of MNPs in aquatic and atmospheric systems, providing critical exposure estimates for ecological risk assessment. Future efforts should integrate microbial community dynamics into multiscale models to better assess the ecological risks of aged MNPs. Multiscale modeling integrates these approaches across molecular, cellular, organismal, and population levels. Machine learning is increasingly used to analyze toxicogenomic data and predict the toxicity of MNPs based on physicochemical properties. In summary, modeling provides mechanistic insights, enables extrapolation across scales, and supports regulatory decision-making.

## 5. Conclusions

This review systematically explores the changes in physical and chemical properties of MNPs before and after aging, the aging mechanisms, influencing factors, and biological toxicity. Environmental factors, such as temperature, particle size, microorganisms, additives, DOM, and inorganic ions, significantly affect the aging process. Aged MNPs have rougher surface morphology, smaller particle size, greater SSA, higher hydrophilicity, and crystallinity. These changes make MNPs more biologically toxic and have stronger interactions with other pollutants, exerting a significant impact on aquatic organisms. Additionally, MNPs can carry pollutants into the food chain, resulting in stronger combined toxicity.

Although the research on MNPs aging has developed rapidly, it still faces many key challenges and knowledge gaps. The following section identifies the key knowledge gaps and unresolved issues in the current research field.

(1) The correlation between laboratory aging simulations and actual wastewater treatment is relatively low. Most studies are conducted in controlled environments (such as constant temperature, a single UV light source, and specific medium), which cannot fully simulate the synergistic or antagonistic effects of various environmental factors (such as heat, mechanical force, light, and biological action) in the natural environment. In particular, under AOPs conditions, UV intensity and oxidant concentrations are typically far higher than those in actual processes, and the differences between the resulting degradation products and real-world conditions require further evaluation.
(2) It remains unknown whether the observed adverse effects stem from the particles themselves or the chemical mixtures in which they were immersed. For toxicity studies, aged particles should be washed and separated from their immersion solution prior to introduction into organisms. Otherwise, it remains unclear whether the increased toxicity of “aged MNPs” stems from more hazardous surfaces or simply from their release of greater quantities of toxic additives (such as PAEs, BPA, or flame retardants) into the test water. Given the critical role of microorganisms in ecosystem functioning, future research should prioritize understanding the long-term impacts of aged MNPs on microbial community dynamics and their ecological consequences.

## Data Availability

No new data were created or analyzed in this study. Data sharing is not applicable to this article.
